# The role of the interferon/JAK-STAT axis in driving islet HLA-I hyperexpression in type 1 diabetes

**DOI:** 10.3389/fendo.2023.1270325

**Published:** 2023-10-06

**Authors:** Mark A. Russell, Sarah J. Richardson, Noel G. Morgan

**Affiliations:** Department of Clinical and Biomedical Sciences, University of Exeter, Exeter, United Kingdom

**Keywords:** HLA-I, STAT1, STAT2, pancreatic islet, type 1 diabetes

## Abstract

The hyperexpression of human leukocyte antigen class I (HLA-I) molecules on pancreatic beta-cells is widely accepted as a hallmark feature of type 1 diabetes pathogenesis. This response is important clinically since it may increase the visibility of beta-cells to autoreactive CD8+ T-cells, thereby accelerating disease progression. In this review, key factors which drive HLA-I hyperexpression will be explored, and their clinical significance examined. It is established that the presence of residual beta-cells is essential for HLA-I hyperexpression by islet cells at all stages of the disease. We suggest that the most likely drivers of this process are interferons released from beta-cells (type I or III interferon; possibly in response to viral infection) or those elaborated from influent, autoreactive immune cells (type II interferon). In both cases, Janus Kinase/Signal Transducer and Activator of Transcription (JAK/STAT) pathways will be activated to induce the downstream expression of interferon stimulated genes. A variety of models have highlighted that HLA-I expression is enhanced in beta-cells in response to interferons, and that STAT1, STAT2 and interferon regulatory factor 9 (IRF9) play key roles in mediating these effects (depending on the species of interferon involved). Importantly, STAT1 expression is elevated in the beta-cells of donors with recent-onset type I diabetes, and this correlates with HLA-I hyperexpression on an islet-by-islet basis. These responses can be replicated *in vitro*, and we consider that chronically elevated STAT1 may have a role in maintaining HLA-I hyperexpression. However, other data have highlighted that STAT2-IRF9 may also be critical to this process. Thus, a better understanding of how these factors regulate HLA-I under chronically stimulated conditions needs to be gathered. Finally, JAK inhibitors can target interferon signaling pathways to diminish HLA-I expression in mouse models. It seems probable that these agents may also be effective in patients; diminishing HLA-I hyperexpression on islets, reducing the visibility of beta-cells to the immune system and ultimately slowing disease progression. The first clinical trials of selective JAK inhibitors are underway, and the outcomes should have important implications for type 1 diabetes clinical management.

## 1 Introduction

Type 1 diabetes (T1D) is an autoimmune disorder which depletes circulating insulin by selective targeting of pancreatic beta-cells leading to their dysfunction and/or loss. This then culminates in the characteristic uncontrolled hyperglycemia seen in this condition. In 2021 it was estimated that approximately 8.4 million individuals were living with T1D worldwide and alarmingly its incidence is still rising year on year in many countries (1, 2). To manage their symptoms, those with T1D are required to administer exogenous insulin subcutaneously multiple times each day. However, despite this leading to improved levels of glycaemia, many individuals still experience poor glycemic control and are at increased risk of developing serious macrovascular and microvascular complications which can ultimately lead to amputation, blindness and kidney failure (3). Given the complex and intense management strategies required to maintain good glycemic control, the cost of T1D to health-care services worldwide is significant (4, 5). As such, much research is focused on identifying alternative treatments which will be beneficial to patients and health-care systems worldwide. These efforts are centered on identifying novel methods to promote beta-cell survival, to replace lost or dysfunctional beta-cells (endogenously or exogenously) and/or to dampen the immune response. Excitingly, this work is beginning to bear fruit since the first drug to delay T1D onset in at risk individuals, Teplizumab (marketed in the USA as Tzield), is now FDA approved and available to certain groups of patients (6, 7).

While much of our knowledge of T1D pathogenesis has come from work examining rodent models of the disease, key insights have increasingly been derived from study of the human pancreas. However, there is a distinct lack of pancreatic tissue available to researchers, in part due to the high risk of surgical complications arising during pancreas biopsies, which placed alongside the lack of clinical benefit arising from this procedure to the individual, means that the risks associated with such biopsies outweigh the benefits, especially for young, otherwise heathy individuals. As such many early studies have derived inferences about the pathogenesis of T1D from the study of a very limited cohort of cadaveric pancreata gathered from specific global regions. To reduce the limitations of working with small cohort sizes from restricted geographical locations there has been a global push to collect larger cohorts of donor pancreas material to better understand T1D pathogenesis. These include the Exeter Archival Diabetes Biobank (EADB; formally the Foulis collection) in the UK, the Network for Pancreatic Organ Donors (nPOD) in the USA, the Human Pancreas Analysis Program (HPAP) in the USA and the Diabetes Virus Detection (DiViD) study in Norway. Critically, pancreata from these initiatives provide a window into the active disease and collaborative efforts across the globe are increasing our collective understanding (8–11). However, to explore the underlying disease mechanisms fully, knowledge gained from these precious resources must be supplemented with studies in rodent and cellular models.

Histological study of pancreas samples has revealed key pathological features of the disease. One of the most striking observations is that a protein complex, which facilitates communication between the immune system and the beta-cell, is highly expressed on islets from donors with T1D but not in those without diabetes. This complex is termed Human Leukocyte Antigen Class I (HLA-I) or the Major Histocompatibility Complex Class I, and its hyperexpression within islets has been described as a ‘hallmark feature’ of the disease (12). In the current review we will explore the evidence for HLA-I hyperexpression and the molecular mechanisms responsible for driving this process in pancreatic beta-cells.

## 2 HLA-I hyperexpression in type 1 diabetes

**Figure 1:**
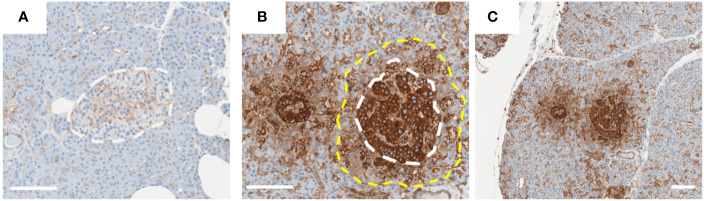
HLA-I hyperexpression in Islets from type 1 diabetes donor. Representative images of islets from donors [**(A)**; 6012] without or [**(B, C)**; 6052] with T1D. Pancreas sections from the nPOD collection were stained for HLA-I using an immunoperoxidase approach. Islets in image **(B)** are magnified from those presented in image **(C)**. White dotted line indicates the approximate edge of the islet, and yellow dotted line the approximate extent of the HLA-I ‘halo’ in the exocrine tissue. Scale bar = 100µm.

HLA-I is an important protein complex that regulates immune-mediated processes to assist in the definition of “self” and “non-self” and is expressed by all nucleated cells in the body [reviewed in (13)]. The complex is composed of a ‘classical’ HLA protein subunit (HLA-A, HLA-B or HLA-C) in association with a second, independently coded, protein, beta-2-microglobulin (B2M). This complex is loaded with short peptide sequences derived from the proteosomal degradation of proteins, in the endoplasmic reticulum, and these are then presented on the surface of the cell where the HLA-I complexes, together with their associated peptides, can interact with CD8+ cytotoxic T-cells as part of the adaptive immune response. Peptides derived from all proteins produced within a cell are presented on HLA-I, including those that are ‘non-self’, for example those produced by any pathogens which may have infected a cell. Typically, specific CD8+ T-cells, via their T-cell receptor (TCR), will identify and selectively destroy infected cells through interaction with the pathogen peptide-loaded HLA-I complexes. However, in the context of type 1 diabetes, this system becomes subverted and works against the beta-cell. Here, a sub-set of proteins normally expressed in the beta-cell is erroneously identified as non-self and an immune response is raised against them. In this context, T-cells targeting beta-cell antigens have been detected in the blood and pancreas of those with T1D (14). As such, HLA-I expressed on beta-cells is central to T1D pathogenesis, but this is further exacerbated since HLA-I levels are markedly elevated in the islets of people with T1D (a feature described as ‘HLA-I hyperexpression’) (**Figure 1**) (12).

The hyperexpression of HLA-I on/in pancreatic islets is described as a ‘Hallmark Feature’ of T1D (12). This important feature of the disease was first described by Bottazzo et al. in 1985 (15) and Foulis et al. in 1987 (16). Since these initial observations, HLA-I hyperexpression has been reported in a variety of independent studies and across multiple cohorts using immunostaining of human pancreas sections (17–19). The validity of these data have been challenged since, in one report, it was suggested that the HLA-I hyperexpression phenomenon is simply an artifact of the staining approaches used (20). In response to this challenge, a comprehensive collaborative study was initiated to explore the phenomenon thoroughly (12). Here, HLA-I hyperexpression was observed in T1D islets using (i) a variety of different staining approaches, (ii) use of separate antibodies recognizing HLA-I, (iii) pancreas preserved using different techniques and (iv) when analyzed in independent laboratories. Moreover, HLA-I mRNA expression (*HLA-A, HLA-B* and *HLA-C*) was also found to be elevated in laser captured islets from these same donors. Finally, B2M was shown to be increased at both the mRNA and protein levels in parallel with HLA-I. Emerging large transcriptomic data from bulk sorted T1D beta-cells similarly revealed raised expression of HLA-I encoding genes and *B2M* (21). Additionally, there is published evidence of elevated expression of non-classical HLA gene products (e.g. HLA-E, HLA-F) on the islets of people with T1D (12, 22), and also of the genes associated with processing, trafficking and loading of peptides onto HLA-I (23). The potential role of elevated non-classical HLA-I in type I diabetes pathogenesis is reviewed elsewhere (24). Taken together, this provides overwhelming evidence that islets from donors with T1D hyperexpress HLA-I. Interestingly, elevated HLA-I is not restricted to just beta-cells, since all other endocrine cells within the islet also hyperexpress the complex (12) although the non-beta-cells are not targeted during the autoimmune attack suggesting that there are specific aspects of antigen presentation which are uniquely aberrant in beta-cells. This has important implications, since the combination of aberrant antigen presentation on HLA-I coupled with markedly elevated levels of HLA-I will render these cells more visible to cytotoxic T-cells and this will potentiate the autoimmune attack, accelerating the disease process.

Understanding the triggers which initiate HLA-I hyperexpression is critical, since this knowledge may aid in the development of novel therapies which delay the onset of T1D and slow the rate of beta-cell destruction by reducing beta-cell visibility to the immune system. In this context, there is one feature which is consistent among all islets which display HLA-I hyperexpression: the presence of residual beta-cells. Islets from people with T1D in which all beta-cells have been destroyed by the autoimmune process do not continue to express elevated HLA-I (12). This is despite the fact, noted above, that HLA-I hyperexpression is not limited solely to beta-cells at earlier stages of the process (12). Thus, it can be concluded that the retention of beta-cells is central to ongoing islet HLA-I hyperexpression in T1D. This raises a second question about the factors involved since it is known that the presence of immune cells within the islets may exacerbate HLA-I hyperexpression. However studies undertaken by Foulis et al, demonstrated that even when individual HLA-I hyper-expressing islets were sectioned throughout their entire volume to seek the presence of immune cells; none were found (16). This therefore supports the notion that HLA-I hyperexpression is induced by the intrinsic production of factors by beta-cells, which can be augmented further if and when immune cells infiltrate the islet. Whatever mechanism is responsible there is a consensus that beta-cells are complicit in their own demise during T1D pathogenesis (25).

To identify the potential drivers of the HLA-I response it is important to understand the point in disease pathogenesis at which HLA-I expression becomes elevated and over what duration this persists. In their initial studies of the T1D pancreas, Foulis and colleagues observed immune cells around many insulin containing islets (insulitis) which displayed elevated HLA-I, however some islets also hyper-expressed HLA-I without evidence of inflammation (16). These data imply that HLA-I hyperexpression may be initiated early, even before the arrival of autoreactive immune cells. In support of this idea, other studies have revealed HLA-I hyperexpression in the islets of autoantibody positive donors who do not have yet have diabetes but are at higher risk of developing the disease (19, 26). Although a positive association between HLA-I expression and CD8+ cell proximity was detected, this was only in samples from T1D donors and not from those who were auto-antibody positive (19). At the other end of the spectrum, islet cell HLA-I hyperexpression may not be sustained indefinitely; even when some residual beta-cells remain. Thus, in work examining long-duration T1D (>11 years), it was noted that HLA-I levels had subsided even in those pancreata that still retain residual insulin containing islets (12). It is tempting to speculate that one of the reasons these beta-cells are preserved is due to this reduction in HLA-I hyperexpression.

While there is likely to be a role for inflammation to regulate HLA-I expression during active T1D, it is clear from the information already presented that there must also be additional triggers which operate at the very early stages of the disease process. In this context, several common viruses, including *Enteroviruses*, have been implicated as putative initiators of T1D. These assertions are based on a variety of strands of evidence including epidemiological data and histopathological studies (27–30). This group of viruses has been shown to be highly trophic for beta-cells, potentially due to the expression of an unusual isoform of the Coxsackie and adenovirus receptor (CAR) by these cells (31). Additional evidence has also been presented revealing the presence of the viral capsid protein, VP1, and increased levels of a raft of viral response proteins (e.g. PKR, MDA5, MxA) in the beta-cells of cadaveric or living donors with T1D (30, 32). Although VP1 can be detected in a few beta-cells of non-diabetic donor pancreata, little evidence of a wide-ranging antiviral response is observed (beyond the induction of PKR expression) in these individuals, suggesting that the beta-cells of T1D donors may respond with unusual vigor to a viral challenge. This concept aligns well with the observation that pathway analyses of T1D-risk genes that are expressed within beta-cells reveals a strong preference for signaling pathways associated with responses to viruses (33). In the following sections we will explore how this inappropriate response to viral infection may drive HLA-I hyperexpression and ultimately T1D, highlighting the signaling mechanisms which may underpin this process.

Careful examination of HLA-I staining of pancreas sections from T1D donors reveals evidence for a possible mechanism which may drive its elevated expression. Study of these samples reveals strong HLA-I staining throughout the islet, but also modestly elevated HLA-I in the exocrine tissue surrounding the islet (sometimes described as a ‘halo’) (**Figure 1**) (12). This HLA-I ‘halo’ gradually diminishes as the distance from the islet increases suggesting that a soluble factor(s) released from within the islet may be responsible. There are two clear candidates which may represent the source of such secreted proteins: (i) virally infected beta-cells, or (ii) immune cells at the islet site. Both of these cell types can produce interferons, a key group of cytokines known to modulate HLA-I expression.

## 3 The role of interferons

**Figure 2:**
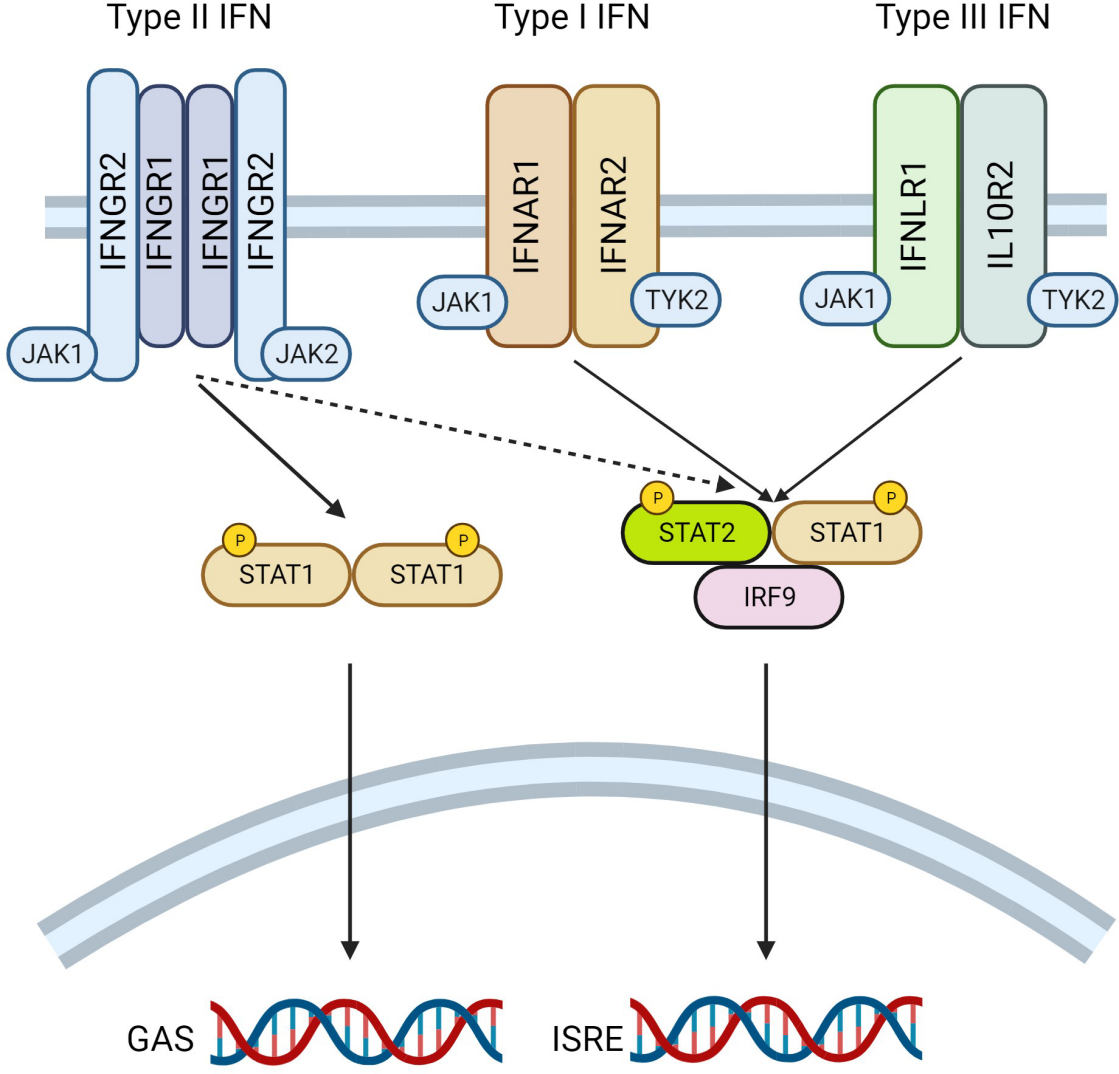
Interferon (IFN) Signalling Mechanisms. A schematic diagram revealing the underlying mechanisms of interferon signalling is presented. IFN stimulate signalling by binding to their cognate cell surface receptor; type I IFN interacts with IFNAR1 and IFNAR2, Type II with IFNGR1 and IFNGR2, whereas type III IFN bind to IFNLR1 and IL10R2. Ligand binding induces receptor dimerization, and the transphosphorylation of Janus Kinases (JAK) associated with the receptor. JAK1 and JAK2 are canonically associated with the type II IFN receptor, with JAK1 and tyrosine kinase 2 (TYK2) bound to type I and type II IFN receptors. Phosphorylation of JAK induces the recruitment and subsequent phosphorylation of signal transducer and activator of transcription (STAT), which interacts with the receptors on their cytoplasmic tails. STATs then dissociate from the receptor, form complexes in the cytosol before moving to the nucleus where they bind to consensus sequences in the DNA. Type II IFN induces the formation of STAT1 homodimers which bind to gamma activated sequences (GAS) in DNA. Alternatively, type I and Type III IFN form STAT1-STAT2 heterodimers, these complex with IFN regulatory factor 9 (IRF9) and bind to IFN sensitive regulatory elements (ISRE) to regulate transcription. Figure generated using Biorender.com.

Interferons (IFN) are a class of cytokine which are involved in a diverse array of biological functions. There are three subclassifications of this family; (i) type I IFN (including IFNα and IFNβ), (ii) type II IFN (IFNγ) and (iii) type III (including IFNλ). Each member functions by binding to a specific cell surface receptor complex to initiate downstream signaling (**Figure 2**) reviewed in (34, 35). Type I IFNs interact with heterodimers comprising IFNa receptor 1 (IFNAR1) and IFNAR2; type II IFNs with the IFNγ receptor 1 (IFNGR1) and IFNGR2; type III IFNs with the interleukin 10 receptor 2 (IL10R2) and IFNλ receptor 1 (IFNLR1). The interaction of IFNs with a receptor monomer induces the dimerization of the receptor complex, and brings Janus Kinases (JAK), already associated with the cytoplasmic tail of each receptor subunit, in closer proximity to one another, leading to their transphosphorylation and enhancing their enzymatic activity. Canonically, type I and III IFN receptors are associated with JAK1 and tyrosine kinase 2 (TYK2), whereas type II IFN receptors interact with JAK1 and JAK2. Increased JAK activity following ligand binding, leads to the recruitment of signal transducer and activator of transcription (STAT) transcription factors, which interact with phospho-tyrosine residues on the cytoplasmic tail of the receptors. JAKs then phosphorylate the recruited STAT molecules on a tyrosine residue in their transactivation domain. This leads to the dissociation of STATs from the receptor complex, dimerization with other STATs in the cytosol, and movement to the nucleus where the STAT complex binds to consensus sequences in DNA to modulate gene transcription. The STAT complexes which mediate these effects differ depending on the species of IFN which initiates signaling. Type I and III IFN induce the formation of STAT1/STAT2 heterodimers which then further complex with interferon regulatory protein 9 (IRF9) to form the IFN stimulated gene factor 3 (ISGF3). ISGF3 binds to IFN stimulated regulatory elements (ISRE) to regulate transcription. Alternatively, type II IFN canonically promotes the generation of STAT1/STAT1 homodimers which interact with gamma activated sequences (GAS) in DNA to modify transcription.

### 3.1 Type I & III IFN

In response to a viral challenge, cells can recognize an infection by detecting intracellular viral replication products (e.g. double-stranded RNA) via endogenous pattern recognition receptors (PRR) and this will stimulate the transcription and elaboration of type I interferons (IFN) (e.g. IFNα and IFNβ) (36), or in certain circumstances type III IFN (e.g. IFNλ) (37). Once released, these cytokines stimulate surrounding cells to upregulate their expression of antiviral interferon stimulates genes (ISG) which aid in reducing the spread of an infection. Genes typically induced include viral-responsive PRRs (e.g. *IFIH1*), key IFN signaling molecules (e.g. *STAT1, STAT2, IRF9*) and genes with direct antiviral activity (e.g. *MX1, OAS1, IFIT1*) reviewed in (38). Thus, this will render cells more able to detect virus, increase their responsiveness to viral infection and enhance their capacity to neutralize the virus, for example through increased degradation of viral nucleic acids (39). In addition to these responses, type 1 IFNs are also reported to stimulate the transcription of classical HLA-I genes in various cell and tissue types, including human pancreatic beta-cells (40, 41). This is likely to be mediated by an ISRE binding site in the proximal promoter of classical HLA-I genes (*HLA-A*, *HLA-B*, *HLA-C*) and *B2M* (42). There are a number of subtypes of type I IFN and it has been shown that these differ in their ability to enhance HLA-I expression in islets (43). Type III IFNs have also been shown to trigger comparable anti-viral responses and HLA-I hyperexpression in beta-cells (44).

Despite the strength of evidence implicating viral infection as a trigger for T1D, it has been challenging to detect direct evidence of IFN release from beta-cells *in situ* in people with T1D. Early studies examining cadaveric pancreas of T1D donors were successful in revealing elevated IFNα mRNA and protein expression in islets, and importantly HLA-I expression in islets was strongly correlated with the presence of IFNα-positive islet cells (45, 46). However, a lack of suitable selective antisera capable of use in tissue sections has meant that more recent studies have not been successful in replicating these findings. Moreover, transcriptomic analysis of bulk sorted beta-cells or laser captured islets did not show an increase in *IFNA* or *IFNL* expression from T1D donors (21, 47). Given that only a minority of beta-cells show evidence of viral infection in T1D, it seems likely that the transcriptional signal from a small number of cells would be too difficult to detect when using these ‘bulk’ approaches, rather a single cell transcriptomic approach might be much more helpful to capture details of type I or type III IFN transcription in these cells.

Although IFNs themselves have been difficult to detect in the islets of T1D donors, there is much more convincing evidence to support a response to type I IFN in these individuals. For example, a type 1 IFN signature was identified in PBMCs before antibody seroconversion from children at risk of T1D (48, 49). A similar signature was detected in laser-captured islets from T1D donors (47), and this was associated within the same islets showing evidence of viral infection of beta-cells (via VP1) and insulitis (50). Moreover, a recent study found a significant overlap between the gene expression changes observed in human islets or beta-cell lines treated with IFNα compared to RNAseq data from purified beta-cells isolated from donors with T1D (22). Many of the genetic signals which are associated with altered T1D risk are found near or within genes either involved in type I IFN signaling (e.g. Tyk2) or are ISGs themselves (e.g. *IFIH1*) (51–53). Rodent models in which viral infection of beta-cells is used to trigger a response similar to T1D again support an important role for type 1 IFN, and in these studies blockade of IFNα signaling can reduce the development of diabetes (54, 55). So, while there is limited direct evidence of type 1 IFN release from virally infected beta-cells during T1D pathogenesis, the supporting evidence presented here strongly suggest that this does occur, and that it may even precede insulitis.

### 3.2 Type II IFN

The infiltration of islets by autoreactive immune cells will be accompanied by an increase in the release of pro-inflammatory cytokines, for example IFNγ, tumor necrosis factor α (TNFα) and interleukin 1β (IL-1β). Chronic exposure of beta-cells to these cytokines is well established to cause impaired insulin secretion and reduced cell viability (56, 57).

While TNFα and IL-1β have not been reported to modify HLA-I expression, treatment of a variety of cell types with IFNγ effectively drives classical *HLA-I* and *B2M* gene expression (58). Rather than being solely mediated by STAT1 homodimers binding to GAS sites, it is thought that this response is also driven through ISRE sites in the proximal promoter of these genes (42, 59). This runs somewhat counter to the previously accepted dogma of canonical type II IFN signaling. However, IFNγ is noted to stimulate ISRE activity, although it is less effective in doing this when compared to IFNα. We recently reported this response in EndoC-βH1 human beta-cells, noting that elevated ISRE activity was only observed after chronic (24h) but not acute (2h) IFNγ treatment (60). It becomes more complex to explain this signaling crosstalk, when considering that IRF9 is required for STATs to bind ISRE (59, 61). However, results from a recent study may provide an explanation (59). In this work, the authors observed that IRF9 knockout in macrophages impacted a surprisingly large number of IFNγ responsive genes. Their data also suggested that STAT2-IRF9 may regulate basal ISG expression, with active STAT1 dimers forming the ISGF3 complex on the DNA rather than in the cytosol. A similar mechanism may be at play in beta-cells but requires exploration.

## 4 STAT1 & STAT2

Since there is compelling evidence that IFNs play a key role in the regulation of beta-cell HLA-I expression, it seems likely that downstream STAT signaling molecules will also play a significant role. In support of this, study of STAT1 in the pancreas of T1D donors reveals that its expression is strongly increased in insulin containing islets, with its levels being much more modest in islets without detectable insulin or in donors without diabetes (12). Furthermore, elevated STAT1 was observed both at the RNA and protein levels in T1D (12, 21). Perhaps surprisingly, similar elevations in STAT2 and IRF9 have not been reported (21). Importantly, additional analyses revealed that STAT1 levels are positively correlated with HLA-I on an islet-by-islet basis (12). These data suggest a functional association between HLA-I and STAT1 in islet cells, but defining the exact nature of this relationship requires additional work. Interestingly, examination of the distribution of STAT1 in islets hyperexpressing HLA-I reveals that while its expression appeared increased in the nucleus of all islet cells, there was a profound elevation in STAT1 levels in the cytosol of beta-cells, a response which was absent in other endocrine cell types (12). This observation is fascinating and highlights that the relationship between STAT1 and HLA-I is not quite as clear when examined at the cellular level and may differ between the islet endocrine cell subtypes.

The elevation in STAT1 has been misinterpreted by some as being indicative of an automatic increase in STAT1 phosphorylation (which may happen to some degree by a mass action effect). However, increases in STAT1 phosphorylation should not be assumed and it must be noted that STAT1 phosphorylation has often not been measured directly in samples displaying an elevation of total STAT1 levels. Rather than indicating increased STAT1 phosphorylation, the rise in STAT1 may be indicative of an increase in total STAT1 activity, since *STAT1* gene expression can be regulated by itself in a positive feedback loop (62). While in the canonical model of the pathway, phosphorylation and increased activity go hand in hand, data from other systems has revealed that unphosphorylated (u)STAT1 can display significant transcriptional activity (63). Thus, the elevated STAT1 observed in T1D islets may have transcriptional relevance even if it is not phosphorylated. In support of this are data from studies undertaken to model the chronic upregulation of STAT1 in human beta-cell lines. In these experiments, chronic treatment with IFNs stimulated an increase in STAT1 expression and the transcription factor remained elevated for more than 96h following IFN removal (60). Although STAT1 phosphorylation was initially stimulated, it quickly reduced to close to unstimulated levels despite the chronic treatment, and moreover, cells became desensitized to additional application of the same or related IFN species (60). Strikingly, these responses were accompanied by a build-up of STAT1 in the cytosol of cells, similar to that seen in T1D islets (12). Thus, under these conditions, beta-cells may be utilising uSTAT1 to drive transcriptional activity of the downstream pathway.

While cellular systems seem to closely model STAT1 in the beta-cells of T1D donors, the same cannot be said about STAT2. In cell models, STAT2 follows an identical pattern to STAT1 and remains elevated after chronic IFN exposure (12), but in the pancreas STAT2 levels are apparently unchanged during T1D pathogenesis (60). Despite this, other data have suggested that STAT2 is central to the control of HLA-I. In one study, silencing of STAT2 in EndoC-βH1 cells partially inhibited IFNα induced HLA-I mRNA and protein expression, whereas, surprisingly, STAT1 knockdown was ineffective (40). Silencing of IRF9 or IRF9 in combination with STAT2 also prevented IFN-induced elevations in HLA-I or other ISGs (40). These data suggest a critical role for STAT2 and IRF9 in regulating HLA-I expression, at least under conditions of acute stimulation. It seems unlikely that STAT1 plays no role in this process since its expression is dynamically altered in T1D and this correlates with HLA-I at the islet level. However, it is worth highlighting that STAT2-IRF9 complexes have been implicated in the prolonged expression of genes following IFN exposure (64). As such, it is clear we need to better understand how these factors cooperate to regulate HLA-I expression in beta-cells, particularly under conditions of chronically elevated STAT1 expression.

## 5 IRF1

Expression of the transcription factor *IRF1* is strongly induced following IFN treatment of cells, and this response has been observed when exposing rodent or human beta-cells to various isoforms of the cytokine (65–67). Its levels are also elevated in some islet cells from donors with T1D, although the pattern of expression does not correlate with a specific endocrine cell subtype or match that previously seen with STAT1 or HLA-I (12, 65). In accord with this, IRF1 mRNA levels were found to be significantly increased in bulk-sorted beta-cells from donors with T1D (21). IRF1 in donor pancreas may be more difficult to observe since *in vitro* experiments in human beta-cell lines have revealed that its expression is transient, diminishing to baseline within as little as 24h after IFN exposure (22). The dynamics of this response are in contrast to STAT1, STAT2 or HLA-I which take at least 8h to increase expression, and then remain elevated for a prolonged period thereafter (60). This observation may be important, since IRF1 is known to work alongside, or in concert with, other transcriptional complexes to promote ISG expression, and as such increased IRF1 may have a role in regulating the expression of HLA-I genes in islets. Thus, it is feasible that during the early stages of IFN exposure in beta-cells these transcription factors work together to boost the initial expression of *STAT1* and *HLA-I* genes. After chronic stimulation, when IRF1 levels are depleted, the increased STAT1 may be sufficient to maintain the expression of the target genes in an elevated state. This hypothesis needs to be tested.

## 6 NLRC5

Although there are various transcription factors which are important in driving HLA-I gene expression, a NOD-like receptor family protein, NOD- LRR- and CARD containing 5 (NLRC5), functions as a key transactivator of HLA-I genes (42). As such, it is plausible that NLRC5 may also be involved in the hyperexpression of HLA-I in T1D islets. In support of this hypothesis, a recent study reported a significant increase in NLRC5 mRNA expression in bulk-sorted beta-cells from T1D organ donors when compared to non-diabetic cells (21). However, this response has not been reported in all cohorts, with other published data showing no change in the protein or mRNA expression of NLRC5 in T1D islets from cadaveric or live donors (12, 20).

The expression of NLRC5 is reported to be elevated in response to the activation of various inflammatory signaling pathways, including following stimulation of cells with type I or type II IFNs (42). In agreement, treatment of human islets, rodent islets or a human beta-cell line with IFNα enhances NLRC5 mRNA and protein expression (68, 69). This elevation remained, at least at the protein level, for up to 72h (68).

Although NLRC5 expression in T1D islets does not consistently correlate with HLA-I expression in all reports, it has recently been revealed that NLRC5 may have an important role in the regulation of interferon responses in human beta-cells (69). This study revealed that knockdown of NLRC5 in EndoC βH1 cells inhibited IFNα responses, including partially attenuating the elevations in HLA-I. Thus, NLRC5 likely remains an important component of HLA-I gene transcription, and the difficulty to detect changes in its expression during T1D pathogenesis possibly reflect sufficient reserves of the protein to respond to changes elsewhere in the pathway [e.g. STAT1 (21)].

## 7 Clinical implications

**Figure 3:**
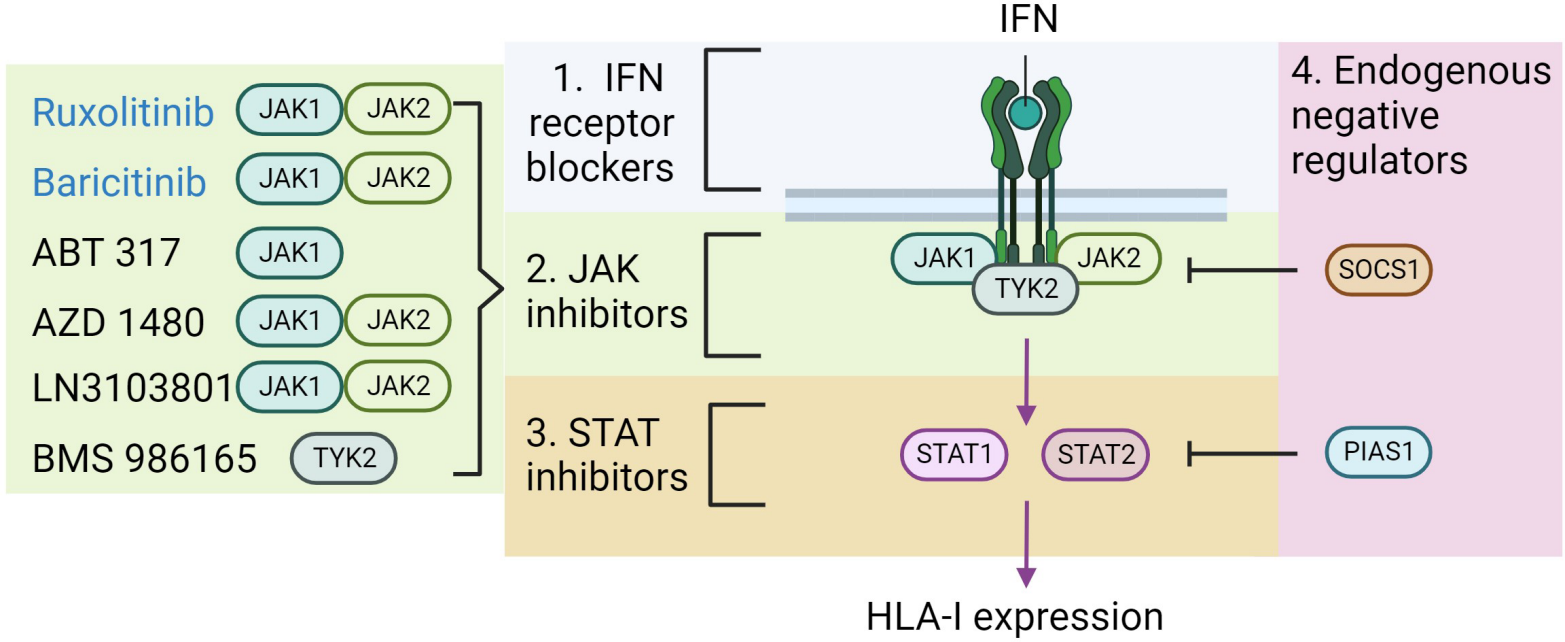
Potential therapeutic strategies to block HLA-I hyper-expression. As previously illustrated IFN binds to its receptor, which increases Janus Kinase (JAK) activity, subsequent signal transducer and activator of transcription (STAT) activity, ultimately promoting HLA-I expression. The diagram highlights the areas where therapeutic strategies might focus to impede this pathway (numbered 1-4). On the left lists the agents which have currently been shown to effectively block IFN induced HLA-I expression in beta-cells, and the proteins that they target. Those in blue text are approved for clinical use in other disease contexts. Figure generated using Biorender.com.

The data presented implicate IFN signaling via STAT1 and STAT2 as key regulators of HLA-I hyperexpression in T1D. As such, clinical modalities which target these pathways will likely be useful in preventing this response and may be an effective treatment for T1D. There are a number of possible clinical targets within this pathway, these include the various IFN receptors, STAT1/2 or the JAKs upstream of STATs (**Figure 3**) (70). There has been very limited success in targeting the IFN receptors or STAT proteins directly. By contrast, an increasing range of JAK inhibitors have been developed or are currently in development, and some of the these are already approved for clinical use in other autoimmune diseases, including rheumatoid arthritis and psoriasis (71, 72).

The early generations of JAK inhibitors were not particularly selective, however in recent years agents have been developed which are much more effective at targeting just one or two of the JAK family of proteins. When thinking about these drugs in the context of canonical IFN signaling, an effective inhibitor will need to target JAK1 or JAK2 to block IFNγ signaling or JAK1 or TYK2 to arrest IFNα. Thus, if both pathways are important in T1D pathogenesis, then targeting JAK1 may be the more effective method to prevent IFN signaling.

There have already been efforts to explore the impact of JAK inhibitors on the development of diabetes in the NOD mouse, a model of T1D (73, 74). The results of these studies have been encouraging with several positive outcomes reported. Notably, the JAK1/2 inhibitor AZD1480 was effective in preventing insulitis in NOD mice, it prevented HLA-I hyperexpression on their islets and crucially reduced the incidence of spontaneous diabetes (73). Encouragingly, the JAK inhibitor also reversed newly diagnosed diabetes in these mice, although these effects were transient (73). A second study using a selective JAK1 inhibitor, ABT 317, reported similar results (74). Importantly, another JAK1/2 inhibitor (LN3103801) was shown to prevent diabetes in NOD mice caused by inhibition of PD-L1 (75). Additional data using Baricitinib (a JAK1/JAK2 inhibitor) in EndoC-βH1 cells showed that this drug could prevent IFNα-induced HLA-I expression (22). There is also proof-of-principal that these drugs may be effective in human diabetes. This anecdotal evidence comes from a case report of a 15 year old boy with diabetes caused by a gain of function STAT1 mutation who was given Ruxolitinib (a JAK1/JAK2 inhibitor) to treat additional symptoms of this disorder including chronic candidiasis and autoimmune enteropathy (76). Strikingly, 12 months after the patient started this treatment, insulin injections were stopped, and he remained euglycemic for a further 12 months.

GWAS studies have identified polymorphisms in the coding sequence of *TYK2* which modify type 1 diabetes risk (52). TYK2 has a central role in mediating type I IFN signaling, and its selective inhibition either via pharmacological agents (BMS-986165) or CRISPR-mediated knockdown in islets generated from human pluripotent stems cells were effective in preventing HLA-I upregulation in response to IFN (77). Similar data were presented after TYK2 knockdown in dispersed human islets and EndoC-βH1 cells (33). These data (summarized in **Table 1**) suggest the real possibility that inhibition of JAKs could provide therapeutic benefit for those with T1D. Indeed, the first clinical trial is underway. The baricitinib in new onset type 1 diabetes (BANDIT) trial, will investigate whether the JAK1/JAK2 inhibitor baricitinib, currently used in the treatment of rheumatoid arthritis and alopecia, can facilitate beta-cell survival in individuals with recent-onset (<100 days) T1D (78). BANDIT is a double-blind, placebo controlled randomized trial and c-peptide will be measured 2h after a mixed meal as the primary outcome. It is estimated to complete in 2024 and will provide a clear indication of the potential of this class of drugs for the treatment of T1D.

**Table 1:** Impact of JAK inhibition on HLA-I expression and diabetes development.

Study	JAK inhibitor	Cells/Models	Impact
Trivedi et al. 2017 (73)	AZD1480 (JAK1/2)	NOD Mice	↓HLA-I on islets ↓insulitis ↓diabetes development Reversed new onset diabetes
Ge et al. 2020 (74)	ABT 317 (JAK 1)	NOD Mice	↓HLA-I on islets ↓insulitis Reversed new onset diabetes
Ge et al. 2022 (75)	LN3103801 (JAK1/2)	NOD Mice	↓HLA-I on islets ↓insulitis ↓anti-PDL1 induced diabetes
Colli et al. 2020 (22)	Baricitinib (JAK1/2)	EndoC-βH1 cells Human Islets	↓HLA-I on beta-cells/islets ↑Cell viability
Chandra et al. 2022 (77)	BMS-986165 (TYK2)	iPSC islets iPSC + TYK2 KD	↓HLA-I on islets ↓T-cell killing of beta-cells
Marroqui et al. 2015 (33)	N/A TYK2 Knockdown	Human Islets EndoC-βH1 cells	↓HLA-I on beta-cells/islets ↑Cell viability
Chaimowitz et al. 2020 (76)	Ruxolitinib (JAK 1/2)	15y Male	Patient euglycemic Insulin therapy discontinued
BANDIT trial (78)	Baricitinib (JAK1/2)	New Onset T1D	Ongoing

While JAK inhibitors are already used clinically, it is important to recognize the potential risks of targeting these proteins. JAKs are ubiquitously expressed and mediators of an array of key biological processes, including hematopoiesis, immune surveillance and cell division. They may also be involved in the cytoprotective actions of agents such as IL-13 in beta-cells (79). Moreover, there are only 4 JAK proteins which regulate these functions. As such, the systemic targeting of JAKs, with inhibitors for example, may inevitably lead to off target effects. It is perhaps surprising therefore, that oral JAK inhibitors are reasonably well tolerated, although several side effects including an increased risk of serious infections (e.g. Herpes zoster reactivation) and anemia are reported (80). There are a few ways in which the risks of these off-target effects can be reduced: (i) identifying the key JAK(s) involved and using appropriate reagents to target these, (ii) generating more specific JAK inhibitors which target only disease relevant JAKs, and (iii) developing methodologies to effectively deliver these reagents to the target cell thereby minimizing their systemic effects. Use of these approaches may provide an effective method to target these drugs to beta-cells, thereby reducing their off-target effects while maintaining their benefits.

## 8 Conclusion

The hyperexpression of HLA-I is now accepted as a hallmark feature of T1D, and it likely plays a significant role in the process of autoimmune-mediated beta-cell destruction. Evidence also points to an important role for IFNs and their underlying JAK/STAT-mediated signaling pathways in driving this process. STAT1, STAT2 and IRF9 have all been implicated in regulating HLA-I expression in response to acute treatment of beta-cells with IFN, with altered STAT1 expression also observed in the islets of donors with T1D. Although many of the players involved in these mechanisms have probably been identified, a clear mechanistic picture of how these proteins work together to drive the response remains to be established. Moreover, it is important to remember that, in the context of T1D, islet cells are likely to be chronically bathed in IFN. Thus, understanding how these mechanisms operate in the context of extended IFN treatment will be important to establish.

JAK inhibitors appear currently to be the best method to target the IFN signaling pathways clinically, and the BANDIT trial will provide some important clarity on the validity of this hypothesis. If BANDIT is successful there is the opportunity for repurposing of JAK inhibitors which are already in clinical use. This will undoubtably simplify the route to get these agents into the diabetes clinic relatively quickly. However, it is important to then not rest on our laurels. The JAK inhibitors available for repurposing may not be those most appropriate for the job (i.e. are they targeting the correct JAKs)?, and we must also consider the most effective methods to deliver these drugs. Moreover, it is important to continue to consider targeting other components of the pathway (**Figure 2**), or even promoting the expression of endogenous negative regulators of these signals [e.g. PIAS1 (81)]. These approaches may provide more cell specific methods to block these signals, thereby reducing unwanted off target effects. Irrespective of the trial results there is sufficient evidence to strongly support the suggestion that targeting of these IFN-mediated pathways will provide a benefit to individuals with T1D.

## Author contributions

MR: Conceptualization, Writing – original draft, Writing – review & editing. SR: Writing – review & editing. NM: Writing – review & editing.
